# Supplementary material for: Constraining PERMANOVA and LDM to within-set comparisons by projection improves the efficiency of analyses of matched sets of microbiome data
Source: Microbiome. 2021 Jun 9;9:133. doi: 10.1186/s40168-021-01034-9 (PMC8191060; doi:10.1186/s40168-021-01034-9)
Supplement: Supplementary file 2 — Additional file 1 Simulation results for the matched-pair data of scenario (1). The LDM on frequency scale (freq), arcsin-root-transformed frequency scale (arcsin), and the omnibus test (omni) are shown. permanovaFL based on the Bray-Curtis (BC), weighted UniFrac (WU), and Hellinger (H) distances are shown. All methods adopted the proposed strategy. [file 40168_2021_1034_MOESM1_ESM.pdf]

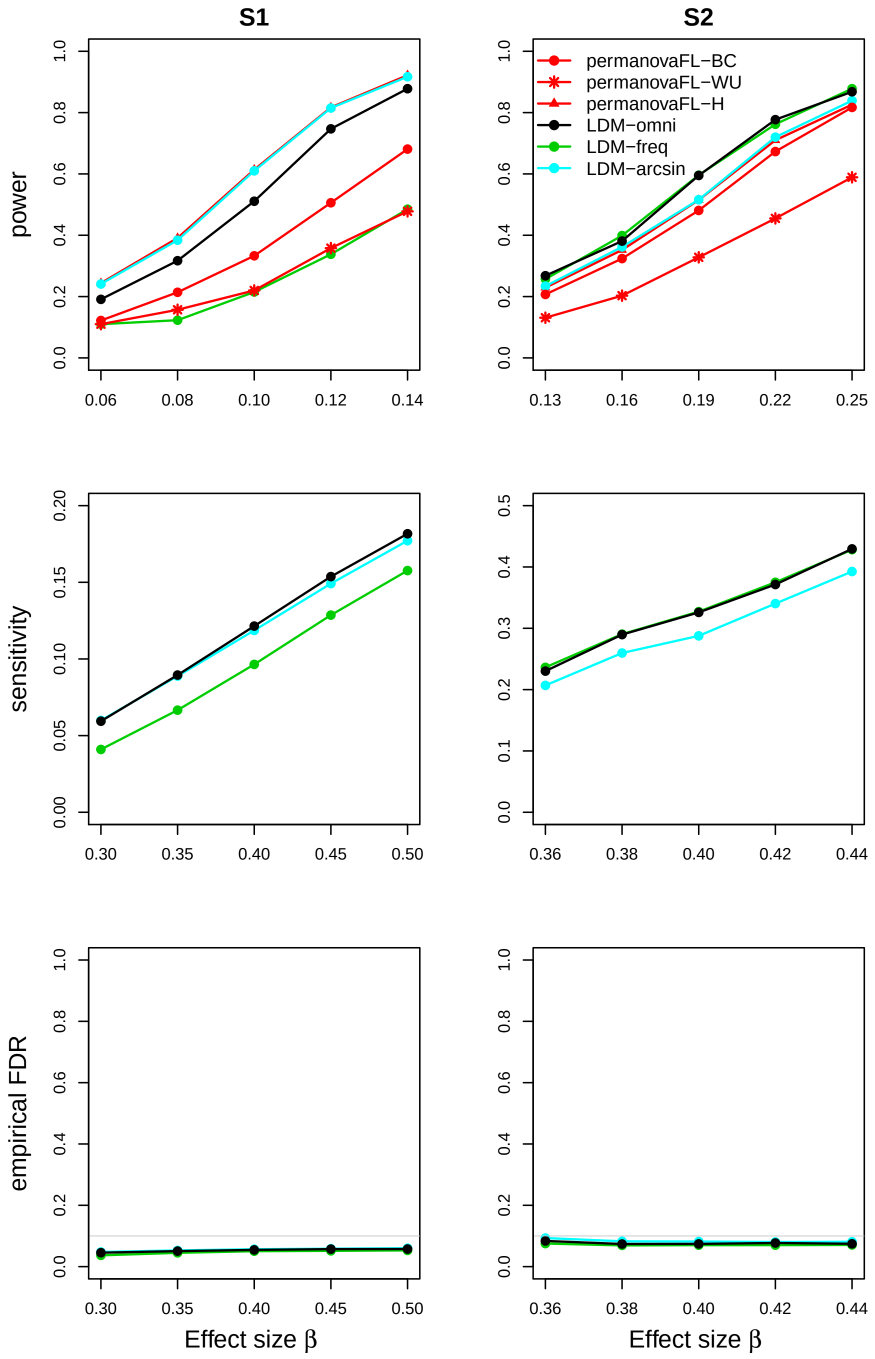

Fig S1. Simulation results for the matched-pair data of scenario (1). The LDM on frequency scale (freq), arcsin-root transformed frequency scale (arcsin), and the omnibus test (omni) are shown. `permanovaFL` based on the Bray-Curtis (BC), weighted UniFrac (WU), and Hellinger (H) distances are shown. All methods adopted the proposed strategy.
